# Combined Transcriptome and Metabolome Analysis of the Quality Change Mechanism of the ‘Pingguoli’ Pear with a Large-Fruited Bud Mutation

**DOI:** 10.3390/plants15081225

**Published:** 2026-04-16

**Authors:** Yuying Li, Xiangyi Wang, Yimo Yang, Junli Wang, Songhao An, Liping Ran, Ju Hu, Yidong Song, Li Cao

**Affiliations:** 1 Department of Horticulture and Landscape Architecture, College of Agriculture, Yanbian University, Yanji 133002, China; 2024050899@ybu.edu.cn (Y.L.); 2024050906@ybu.edu.cn (X.W.); yangyimo0918@163.com (Y.Y.); 2025050937@ybu.edu.cn (J.W.); ansonghao@ybu.edu.cn (S.A.); 0000004092@ybu.edu.cn (L.R.); 0000008971@ybu.edu.cn (J.H.); 2 Jilin Longjing Apple Pear Science and Technology Courtyard, Yanbian University, Yanji 133002, China; 3 Yanbian Prefecture Agricultural Biological Resources and Rural Energy Management Station, Yanji 133002, China; yidong1023@outlook.com

**Keywords:** ‘Pingguoli’ pear, bud mutation, fruit quality, transcriptome, metabolome

## Abstract

The ‘Pingguoli’ pear (*Pyrus pyrifolia* cv. Pingguoli) has a cultivation history spanning nearly one hundred years. Bud mutation selection is an important breeding method for the ‘Pingguoli’ pear. In this study, high-throughput sequencing technology (RNA-Seq) and non-targeted metabolomics (LC-MS/MS) were used to analyze the large-fruited bud mutation line (LFS) and normal type (NTF) of the ‘Pingguoli’ pear during the cell division (G1), rapid growth (G2), and mature stages (G3) of the fruit. The results showed that LFS exhibited a 46.32% increase in average single fruit weight (383.01 ± 54.72 g vs. 261.76 ± 10.79 g, *p* < 0.01) and a 19.10% decrease in soluble solids content (12.70 ± 0.94% vs. 15.40 ± 2.06%, *p* < 0.05) compared to NTF. Compared with the NTF, the content of total phenols and total flavonoids and the activity of antioxidant enzymes in the LFS fruits were significantly higher, while the contents of soluble sugar, reducing sugar, and soluble protein were significantly lower. Transcriptome analysis revealed that key metabolic pathways—including pentose and glucuronate interconversions, starch and sucrose metabolism, and cutin, suberine, and wax biosynthesis—were significantly enriched between NTF and LFS. These pathways may contain the specific differentially expressed genes (e.g., those involved in sugar metabolism and wax biosynthesis) identified as potential regulators of fruit size, appearance, and nutritional quality in the LFS. LC-MS/MS analysis identified key differentially accumulated metabolites, including L-arginine, caffeic acid, L-cysteine, pyridoxamine 5′-phosphate, adenosine-5′-phosphosulfate, neopentolactone D, chlorogenic acid, and gluconic acid, which are directly associated with the nutritional and antioxidant differences between LFS and NTF. The genes most related to metabolites in the three different developmental periods of the LFS and NTF were identified through combined analysis. These results provide insights for further research on bud mutation breeding and the quality formation mechanism of ‘Pingguoli’ pears.

## 1. Introduction

The ‘Pingguoli’ pear (*Pyrus pyrifolia* cv. Pingguoli) is a traditional fruit in China belonging to the genus Pyrus in the Rosaceae family [1]. The ‘Pingguoli’ pear has a cultivation history spanning nearly a hundred years. It is widely planted in Yanbian Korean Autonomous Prefecture, Jilin Province, China, with a total area of 550 hectares and an annual output of 100,000 tons. It is one of the high-quality agricultural specialties of Yanbian [2,3]. The ‘Pingguoli’ pear is rich in cellulose and water, which can clear and moisten the lungs, eliminate phlegm, and relieve cough. This variety can also promote intestinal peristalsis and has a diuretic effect [4]. With the continuous evolution of and increase in pear consumption, consumers’ demands for pear fruits in terms of color and size are becoming increasingly specific. Fruit size is one of the most important commercial characteristics of fruits and has always been valued by breeders. Bud mutation selection is the most likely way of producing large fruit varieties.

Bud mutation selection refers to a kind of somatic mutation that occurs as a result of the natural variation in genetic material in the meristematic tissue of plant buds [5]. Bud changes on fruit trees can endow traditional varieties with new traits, such as variations in fruit size [6], color [7], flavor [8], and changes in the maturity cycle [9]. Bud mutation breeding is simple and easy to master, allows for the development of superior varieties, and continuously provides new germplasm resources. Research on bud mutations in fruit trees has mainly focused on chromosomal structural and numerical variations, the insertion of reverse-transcription transposons, DNA methylation, differences in gene structure and expression, etc. [10,11]. In the process of breeding new varieties of the ‘Pingguoli’ pear, bud mutation selection is the main way of improving the variety. Therefore, conducting research on the bud mutation generation mechanisms in the ‘Pingguoli’ pear is critical, as it can help us to screen out variations with better traits from existing superior varieties to further improve this variety.

Chromosomal ploidy leads to cell enlargement [12], which can cause the flowers, leaves, and fruits of plants to enlarge. Previous studies have shown that a new bud variety with the Cui Guan pear as the parent had significantly larger fruit [13]. The main soluble sugars in the fruit of both the NTF and the large-fruit-type bud transformation variety of ‘Pingguoli’ pear include fructose, glucose, sorbitol, and sucrose [14]. Neither the NTF nor the large-fruit-type sprouted ‘Pingguoli’ pear produce citric acid during the cell division period, and this variety has the highest content of quinic acid [15]. The malic acid content of the bud variant ‘Pingguoli’ pear is the highest in the mature stage, and the citric acid content in the NTF is also the highest in the mature stage [15,16]. In both the NTF and bud variant type of ‘Pingguoli’ pear, the shikimic acid content is the highest during the cell division period [15,17]. Despite these results, there is a lack of systematic comparison of and research on the quality formation mechanism of large-fruit-shaped bud mutation variety and normal ‘Pingguoli’ pears at different development stages.

Despite extensive research on pear fruit quality, the molecular mechanisms underlying bud mutation-induced changes in fruit size and quality remain poorly understood, particularly in ‘Pingguoli’ pear. In this study, we employed high-throughput sequencing (RNA-Seq) and non-targeted metabolomics (LC-MS/MS) to investigate the molecular mechanisms underlying fruit quality variation in a large-fruited bud mutant (LFS) of ‘Pingguoli’ pear compared to the normal type (NTF) across three key developmental stages (cell division, rapid growth, and maturation). By integrating transcriptomic and metabolomic data, we aimed to identify key pathways and gene-metabolite networks governing the trade-off between increased fruit size and altered sugar, phenolic, and antioxidant profiles in LFS. Specifically, we focused on pathways such as pentose and glucuronate interconversions (involved in cell wall biosynthesis), starch and sucrose metabolism (directly linked to sugar accumulation), and phenylpropanoid biosynthesis (associated with phenolic compounds and antioxidant capacity). Understanding how these pathways are coordinately regulated during bud mutation will not only reveal the molecular basis of fruit quality formation in ‘Pingguoli’ pear but also provide a theoretical framework for breeding improved varieties through bud mutation selection in other horticultural crops.

## 2. Results

### 2.1. Appearance Quality and Soluble Solids Observed During the Development of ‘Pingguoli’ Pear

**Figure 1:**
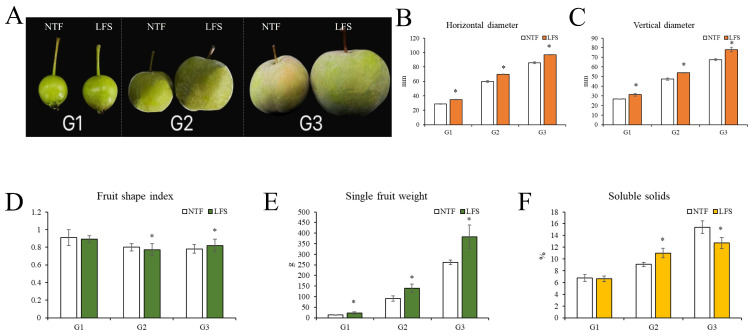
(**A**) Two kinds of ‘Pingguoli’ development states at different stages. The left side of the figure represents ‘NTF’ and the right side represents the ‘LFS’ cell division phase (G1 phase), rapid growth phase (G2 phase), maturation phase (G3 phase). (**B**–**F**) Determination of various indices of two types of ‘Pingguoli’ in different periods ((**B**) horizontal diameter; (**C**) vertical diameter; (**D**) fruit shape index; (**E**) single fruit weight; (**F**) soluble solids). LFS, large-fruited bud mutation lines; NTF, normal type ‘Pingguoli’ pear. G1, fruit cell division stage; G2, the rapid growth stage; G3, the maturity stage. “*” indicates statistically significant differences between the two groups.

During fruit development in the ‘Pingguoli’ pear, both the transverse and longitudinal diameters of the NTF and LFS exhibited a significant upward trend, and the transverse and longitudinal diameters of the LFS were larger than those of the NTF (Figure 1A–C). The fruit shape index of the NTF showed a linear downward trend, while the LFS showed a trend of first decreasing and then increasing (Figure 1D). Generally speaking, within the range of 0.8 to 0.9, the fruit was nearly circular, while within the range of 0.8 to 0.6, it adopted an approximately oval shape. The results showed that the average single fruit weight of LFS (383.01 ± 54.72 g) was 46.32% heavier than that of NTF (261.76 ± 10.79 g, *p* < 0.01). During fruit development, the individual fruit weight of the LFS was always greater than that of the NTF, and it increased by 121.25 g when full maturity was reached (Figure 1E). In this experiment, the content of soluble solids was measured starting from 30 days after fruit harvest (Figure 1F). Both the NTF and LFS showed an upward trend. When the pears were picked while not yet ripe, the content of soluble solids was relatively low, with the NTF exhibiting a higher value than the LFS, but not significantly. During the rapid growth period (G2), fruit growth began to accelerate, and the content of soluble solids for the NTF was lower than that for the LFS, with a difference of 17.55%. At the mature stage (G3), the soluble solids content (SSC) of NTF (15.40 ± 2.06%) was 21.20% higher than that of LFS (12.70 ± 0.94%, *p* < 0.05), indicating a sweeter taste in NTF. In production, the content of soluble solids is often used to compare the sugar content of fruit. Generally, pears with a soluble solids content of over 13% tend to be sweeter. Therefore, the NTF ‘Pingguoli’ pear has a relatively high soluble solids content and a good taste.

### 2.2. The Physicochemical Index During ‘Pingguoli’ Pear Development

**Figure 2:**
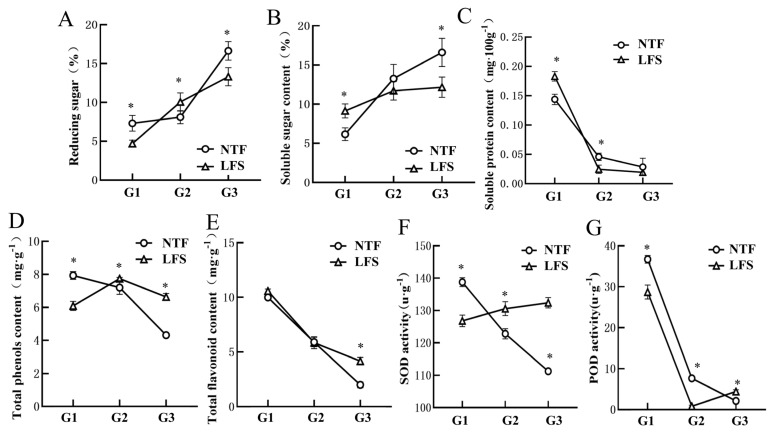
Two types of reducing sugars (**A**) and soluble sugars (**B**) in ‘Pingguoli’ at different development stages. (**C**) Soluble protein of two types ‘Pingguoli’ at different developmental stages. Two types of phenols (**D**) and flavonoid (**E**) content in ‘Pingguoli’ at different stages of development. Antioxidant enzyme activities of SOD (**F**) and POD (**G**) of ‘Pingguoli’ at different developmental stages. LFS, large-fruited bud mutation lines; NTF, normal type ‘Pingguoli’ pear. G1, fruit cell division stage; G2, the rapid growth stage; G3, the maturity stage. “*” indicates statistically significant differences between groups.

During the growth and development of the fruit, the patterns of variation in the reducing sugar and soluble sugar contents in the NTF and LFS ‘Pingguoli’ pear were basically the same. The reducing sugar content of the LFS in G1 decreased by 0.36% relative to the NTF (Figure 2A), and was slightly higher than that of NTF in G2. However, after the fruit matured, the content in the NTF was 0.25% higher than that in the LFS and reached the maximum value. There were also certain differences in soluble sugar content between the NTF and LFS, and in the G2 and G3 phases, the accumulation of reducing sugar in the mature fruit of the NTF was relatively high (Figure 2B). In the G1 phase, the soluble sugar content of the LFS was 0.49% higher than that of the NTF. In the G3 phase, a large amount of soluble sugar accumulated in the fruit, and the content in the NTF was significantly higher than that in the LFS and reached a maximum (16.70%). Furthermore, the soluble protein content in both the NTF and LFS showed a decreasing trend at different developmental stages (Figure 2C).

Throughout the entire fruit development process, the total phenol content of the NTF showed a decreasing trend, while that of the LFS pear showed a trend of first increasing and then decreasing (Figure 2D). The results of an analysis of the total flavonoid content revealed a linear downward trend in both the NTF and LFS (Figure 2E). The SOD enzyme activity in the NTF showed a linear downward trend, while the SOD enzyme activity in the LFS showed a linear upward trend (Figure 2F). During the G2 and G3 phases, the SOD enzyme activity of the LFS was significantly higher than that of the NTF (*p* < 0.05). The activity of the POD enzyme showed a linear downward trend in the NTF, while it showed a trend of first decreasing and then increasing in the LFS (Figure 2G).

### 2.3. Transcriptome Analysis of Differentially Expressed Genes (DEGs)

**Figure 3:**
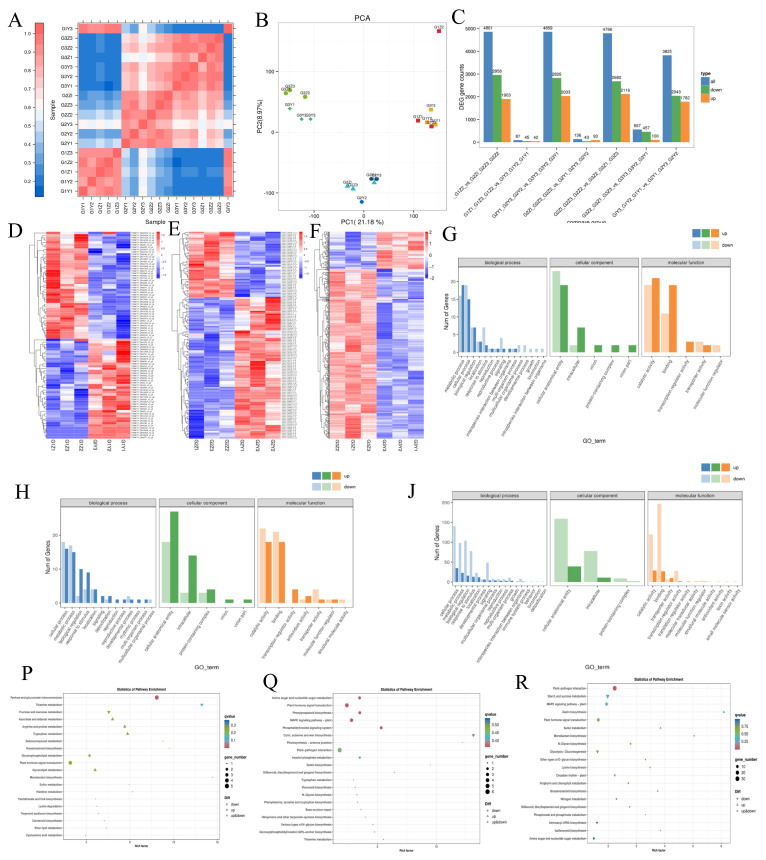
(**A**) Sample correlation heat map. (**B**) Sample principal component analysis. (**C**) Statistical histogram of differential genes. (**D**–**F**) Difference comparison clustering heat map. (**D**) is G1Z vs. G1Y, (**E**) is G2Z vs. G2Y, (**F**) is G3Z vs. G3Y. (**G**,**H**,**J**) Statistical chart of GO annotation classification for differently expressed genes. (**G**) is G1Z vs. G1Y, (**H**) is G2Z vs. G2Y, (**J**) is G3Z vs. G3Y. (**P**–**R**), KEGG pathway enrichment of differently expressed gene. (**P**) is G1Z vs. G1Y, (**Q**) is G2Z vs. G2Y, (**R**) is G3Z vs. G3Y. Each column in the heat map represents different samples, and each row represents different genes. The color indicates that the gene expression level in the sample is Log10 (FPKM + 0.000001). The redder the color, the higher the expression level; the bluer the color, the lower the expression level. The higher the enrichment factor in the KEGG plot, the higher the content of differentially expressed genes in the metabolic pathway. The lower the *p*-value, the more reliable the enrichment significance of differentially expressed genes in this pathway. G1, fruit cell division stage; G2, the rapid growth stage; G3, the maturity stage.

In this experiment, 18 libraries were constructed in three different periods for the ‘Pingguoli’ pear. After sequencing and quality control, a total of 111.32 Gb of clean data was obtained. The Q30 base percentage of all samples was above 92.50%, and all sequencing error rates were within 0.1%, indicating that the sequencing results met the quality requirements (Table S1) for subsequent analysis to be conducted. The results of the sample-to-sample correlation analysis indicated that the correlations among samples G1Y1, G1Y2, G1Z1, G1Z2, and G1Z3 were relatively high, while the correlations between samples in the G1 and G3 phases were low. This finding suggests that the intra-group correlations were higher in each group (Figure 3A). The results of the principal component analysis (PCA) indicated that PC1 contributed 21.18%, while PC2 contributed 8.97%, thus, the sum of the contributions of the two principal components was 30.15% (Figure 3B). The repeatability within each group was relatively good. G3Y1, G3Y2, and G3Y3 were relatively concentrated, while G1Z1, G1Z2, and G1Z3 were slight outliers (Figure 3B).

A differential expression analysis of genes between the NTF and LFS varieties was conducted in this experiment. The results indicate that there were 87 DEGs in G1, among which 42 were upregulated and 45 were downregulated. There were 136 DEGs in the G2 phase, among which 93 were upregulated and 43 were downregulated. There were 557 DEGs in the G3 phase, among which 100 were upregulated and 457 were downregulated (Figure 3C). Then, we analyzed the hierarchical clustering of the expression profiles of each DEG in the G1, G2, and G3 phases in the NTF and LFS (Figure 3D–F). To explore the causes of the large-fruit-type bud mutation in the ‘Pingguoli’ pear, in this experiment, the DEGs in the G1, G2, and G3 phases in the NTF and LFS varieties were combined for GO functional analysis. The results revealed that these DEGs mainly involved metabolic processes and cellular processes, and their gene expression levels were significantly upregulated, while the genes related to the response stimuli showed a trend of downregulation (Figure 3G,H,J).

KEGG analysis showed that during G1, DEGs were significantly enriched in the pentose and glucuronate interconversion pathways. Among these, TRINITY_DN4339_c0_g1 (encoding a putative UDP-glucuronate decarboxylase) was downregulated in LFS, suggesting altered cell wall polysaccharide metabolism that may contribute to enhanced cell expansion potential. The DEGs in G2 were mainly significantly enriched in phenylpropanoid biosynthesis; plant hormone signal transduction (TRINITY_DN14217_c0_g1, TRINITY_DN15128_c0_g1, TRINITY_DN15660_c0_g1, TRINITY_DN3732_c0_g1, TRINITY_DN6478_c0_g1, etc.); the MAPK signaling pathway—plant (TRINITY_DN14217_c0_g1, TRINITY_DN15128_c0_g1, TRINITY_DN16188_c0_g1, and TRINITY_DN25027_c0_g1); cutin, suberine, and wax biosynthesis (TRINITY_DN5895_c0_g1 and TRINITY_DN5895_c0_g2); and the phosphatidylinositol signaling system (TRINITY_DN15303_c0_g1, TRINITY_DN16188_c0_g1, and TRINITY_DN5759_c0_g1). In G3, DEGs were mainly enriched in starch and sucrose metabolism, a pathway directly linked to fruit sweetness. Candidate genes such as TRINITY_DN10365_c0_g1, TRINITY_DN11876_c0_g1, TRINITY_DN12446_c0_g2, and TRINITY_DN1531_c2_g2 were identified as potential regulators of sugar accumulation in LFS. The MAPK signaling pathway—plant included TRINITY_DN10451_c0_g2, TRINITY_DN10783_c0_g1, TRINITY_DN130_c0_g1, etc.), zeatin biosynthesis (TRINITY_DN17123_c0_g1, TRINITY_DN20588_c0_g1, and TRINITY_DN7019_c0_g2), and plant hormone signal transduction (TRINITY_DN10024_c0_g1, TRINITY_DN10451_c0_g2, TRINITY_DN1168_c0_g1, etc.).

### 2.4. Metabolomic Analysis of Differentially Accumulated Metabolites (DAMs)

**Figure 4:**
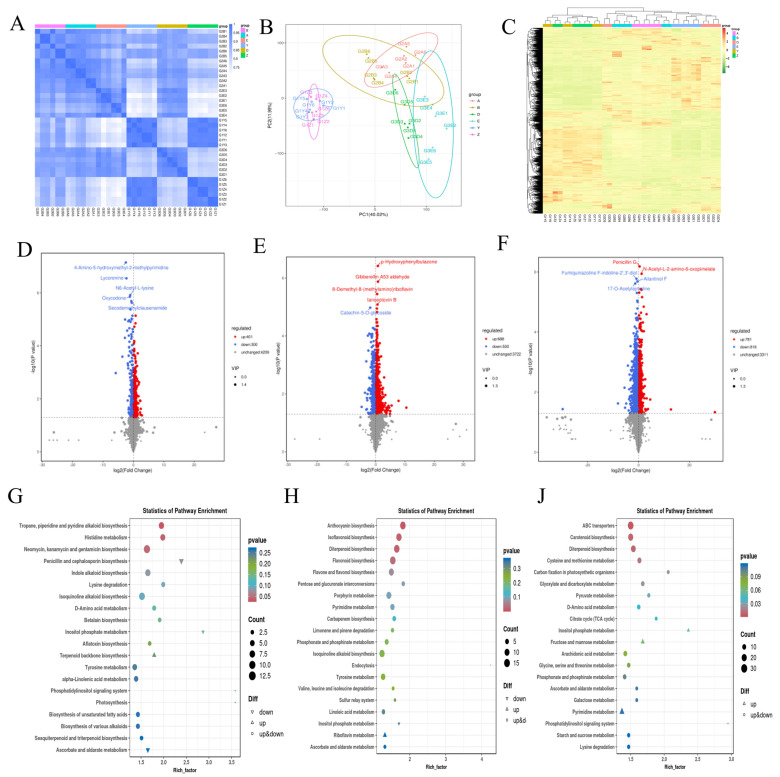
(**A**) Inter-sample correlation plots. (**B**) Metabolite principal component analysis graph. (**C**) Statistical histogram of differential metabolites. (**D**–**F**) Differential metabolite volcano map of G1Z vs. G1Y (**D**), G2A vs. G2B (**E**), and G3D vs. G3E (**F**). (**G**,**H**,**J**) Differential metabolite KEGG enrichment map of G1Z vs. G1Y (**G**), G2A vs. G2B (**H**), G3D vs. G3E (**J**). LFS, large-fruited bud mutation lines; NTF, normal type ‘Pingguoli’ pear. G1, fruit cell division stage; G2, the rapid growth stage; G3, the maturity stage.

The correlation between the six samples of the NTF and LFS ‘Pingguoli’ pear was strong (Figure 4A). During the G1 phase, the correlation between the NTF and LFS was strong, while the correlation between G2 and G3 was weak. The results of the principal component analysis (PCA) of metabolic compounds indicated that the NTF and LFS samples were significantly different. The total variance of PC1 was 40.20%, the total variance of PC2 was 11.99%, and the sum of the two principal components was 52.19% (Figure 4B). A total of 21,332 peaks were detected in this experiment using LC-MS/MS, among which 4910 metabolites were annotated. In this experiment, hierarchical clustering was utilized to analyze the accumulation patterns of these metabolic compounds in different samples (Figure 4C). The results of a volcanic plot analysis of the DAMs indicated that there was a total of 701 DAMs of the NTF and LFS during cell division (G1Z vs. G1Y), among which 401 metabolites were upregulated and 300 metabolites were downregulated (Figure 4D). A total of 1188 types of DAMs were identified during the rapid-growth phase (G2A vs. G2B), among which 688 types were upregulated and 500 were downregulated (Figure 4E). A total of 1599 types of DAMs were identified in the mature stage (G3D vs. G3E), among which 781 types were upregulated and 818 types were downregulated (Figure 4F).

Furthermore, five representative upregulated DAMs were identified in the G1 phase (caffeine, gypenoside A, trans-heptaprenyl diphosphate synthase, vitamin A, and cimigenoside), along with six significantly downregulated DAMs (costunolide, N1, N8-diacetylspermidine hydrochloride, 3α-hydroxy-5α-ethylenedione 17-ketone, 21, 22-dipropionylaniline, 16-hydroxyhexadecanoic acid, and (2R-3R)-3-methylornithine-N6-lysine). During G2, key DAMs such as gluconic acid, tyrosine, and methionine were significantly accumulated in LFS, reflecting enhanced organic acid and amino acid metabolism that may compete with sugar accumulation for carbon skeletons. In G3, metabolites including caffeine and neopentolactone D were upregulated in LFS, pointing to the activation of secondary metabolism and stress defense pathways at the expense of primary sugar accumulation. The results of the KEGG enrichment analysis indicate that the DAMs in G1 were mainly enriched in tropane, piperidine, and pyridine alkaloid biosynthesis, photosynthesis, histidine metabolism, ascorbate and aldarate metabolism, and neomycin, kanamycin, and gentamicin biosynthesis (Figure 4G). The DAMs in G2 were mainly enriched in isoflavonoid biosynthesis, diterpenoid biosynthesis, flavonoid biosynthesis, glycolysis/gluconeogenesis, and anthocyanin biosynthesis (Figure 4H). The DAMs in G3 were mainly enriched in ABC transporters, carotenoid biosynthesis, diterpenoid biosynthesis, cysteine and methionine metabolism, carbon fixation in photosynthetic organisms, and the citrate cycle (TCA cycle) (Figure 4J).

### 2.5. Combined Analysis of the Transcriptome and Metabolome

The O2PLS model was established through RNA-Seq and LC-MS/MS data, and a correlation analysis was conducted (Figure S1A–C). The results show that the NTF and LFS of the ‘Pingguoli’ pear exhibited representative DAM packages in G1, including soyasaponin VI, narbomycin, biochanin A-beta-D-glucoside, (S)-chiral alcohol, 5,7-dihydroxy-4-phenylcoumarin, bacteriopheophytin, 4′, 6-dihydroxy-5, 7-dimethoxyflavanone, tetrangomycin, pseudobaptigenin, thymidine, verruculogen, oleoylethanolamide, 6′-dehydro-6′-oxoparomamine, L-alpha-glutamyl-L-tyrosine, and CDP-glucose. Correspondingly, the representative DEGs were TRINITY_DN29786_c0_g1, TRINITY_DN280_c0_g2, TRINITY_DN5047_c0_g1, TRINITY_DN9636_c0_g1, TRINITY_DN410_c0_g1, TRINITY_DN14278_c0_g1, TRINITY_DN2308_c0_g1, etc. The results suggest coordinated regulation of secondary metabolism in LFS. The DAMs identified in G2 include 4-hydroxyphenylacetaldehyde, gentiolactone, 3-(3′-methylthio) propylmalic acid, 2-indanone, 6-O-p-methyl-N-deacetylisoipecoside aglycon, 2-hydroxy-2,3-dihydrogenistein, 6-O-p-methoxycinnamoylcataipol, trehalose 6-phosphate, guanine, pyridoxamine 5′-phosphate, anamorelin, triethyl citrate, L-homoserine, hispidin, and sesaminol 2-O-beta-D-glucoside. Correspondingly, the key DEGs were TRINITY_DN4015_c0_g1, TRINITY_DN4901_c0_g1, TRINITY_DN1251_c0_g1, TRINITY_DN14616_c0_g1, TRINITY_DN15442_c0_g2, TRINITY_DN47847_c0_g1, TRINITY_DN22770_c0_g1, TRINITY_DN10579_c0_g1, etc. The results highlight candidate regulatory modules during rapid growth. The DAMs identified in G3 include rhodopinal glucoside, N-hydroxy-L-valine, neopterin, terpendole, deoxycytidine, aspartame, 2-dehydro-3-deoxy-D-arabino-heptonate 7-phosphate, 14(R)-lipoxin B4, 1-(sn-Glycero-3-phospho)-1D-myo-inositol, 2,6-dimethylbenzoquinone, gly, tyr, asn, salicylacyl glucuronide, genistein 7-O-beta-D-glucoside, piscidic acid, and 7-mercaptoheptanoylthreonine. The key DEGs identified in G3 include TRINITY_DN8997_c0_g1, TRINITY_DN3803_c0_g1, TRINITY_DN6882_c0_g1, TRINITY_DN12096_c0_g1, TRINITY_DN8262_c0_g2, TRINITY_DN32721_c0_g1, TRINITY_DN25983_c0_g1, and TRINITY_DN204_c0_g1. These results provide candidate targets for understanding mature fruit quality in LFS.

The nine-quadrant correlation plot shows the differential expression of the ‘Pingguoli’ pear gene and its metabolites in the NTF and LFS (Figure S1D–F). In the G1 phase, glucovanillin was positively correlated with 38 genes and negatively correlated with 36 genes; cinnamyl anthranilate was positively correlated with 43 genes and negatively correlated with 29 genes; and isohamnetin (3-methylquercetin) was positively correlated with 42 genes and negatively correlated with 28 genes. In the G2 phase, 4-pyridoxic acid was positively correlated with 78 genes and negatively correlated with 23 genes; 1,4-naphthoquinone was positively correlated with 184 genes and negatively correlated with 31 genes; and bisphosphate was positively correlated with 29 genes and negatively correlated with 20 genes. In the G3 phase, trans-3-hydroxy-L-proline was positively correlated with 39 genes and negatively correlated with 100 genes; naringenin 7-O-rutinoside was positively correlated with 11 genes and negatively correlated with 2 genes; and 2′-deoxyinosine was positively correlated with 28 genes and negatively correlated with 9 genes.

We compared the pathways involving the genes in the transcriptome and metabolites in the metabolome, determined the number of jointly involved pathways, and constructed a corresponding Venn diagram. The results showed that a total of 245 pathways were detected in the groups of the three different growth and development stages of the NTF and LFS; namely, Z vs. Y (G1 phase), A vs. B (G2 phase), and D vs. E (G3 phase). In the Z vs. Y comparison group, 27 KEGG pathways involving both genes and metabolites were identified. There were 23 pathways in the A vs. B comparison group, while there were pathways in the D vs. E comparison group (Figure S1G,H,J).

### 2.6. Combined KEGG and Correlation Network Analysis of RNA-Seq and LC-MS/MS

**Figure 5:**
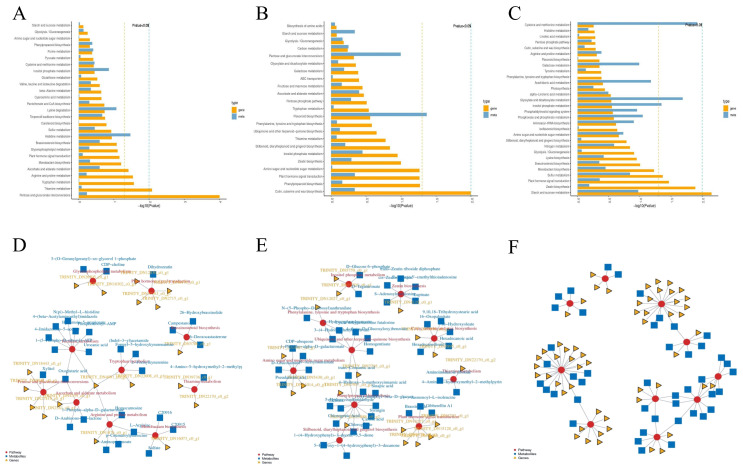
(**A**–**C**) Histogram of differential gene/metabolite KEGG enrichment. (**A**) G1Z vs. G1Y; (**B**) G2A vs. G2B; (**C**) G3D vs. G3E. (**D**–**F**) Pathway vs. differential gene/metabolite kgml network diagram. (**D**) G1Z vs. G1Y; (**E**) G2A vs. G2B; (**F**) G3D vs. G3E. LFS, large-fruited bud mutation lines; NTF, normal type ‘Pingguoli’ pear. G1, fruit cell division stage; G2, the rapid growth stage; G3, the maturity stage.

Joint KEGG analysis confirmed that pentose and glucuronate interconversions (G1), cutin, suberine, and wax biosynthesis (G2), and starch and sucrose metabolism (G3) were the most significantly co-enriched pathways. These pathways represent key metabolic nodes where gene expression changes directly impact metabolite profiles, ultimately driving the phenotypic divergence between LFS and NTF (Figure 5A), followed by thiamine metabolism (*p* < 0.01), tryptophan metabolism, arginine and proline metabolism, and ascorbic acid and alginate metabolism (*p* < 0.05). In G2, cutin, suberine, and wax biosynthesis and flavonoid biosynthesis emerged as the most significantly co-enriched pathways, suggesting their roles in secondary metabolism and stress response during rapid growth (Figure 5B). In G3, starch and sucrose metabolism was the most significantly co-enriched pathway, directly linking gene expression changes to sugar accumulation and sweetness in LFS fruit, followed by zeaxanthin biosynthesis, plant hormone signal transduction, carotenoid biosynthesis, glyoxylic acid and dicarboxylic acid metabolism, carbon fixation in photosynthetic organisms, and sulfur metabolism (*p* < 0.05) (Figure 5C).

In this experiment, a correlation network analysis was conducted on these important DAMS and DEGs. In the NTF and LFS, we identified 22 genes and 31 metabolites with significant differences in G1 (Figure 5D) including coumaryl putrescine, ribose phosphate -AMP, dihydroyulin, xylitol, campesterol, L-arginine, homocarnosine, 3-acetamide, gluconic acid oxide, methyl 5-aminovalerate, 6-deoxybrassinolone, sulfate, 1-(5-phosphate-D-ribonucleic acid)-ATP, TRINITY_DN16073_c0_g1, TRINITY_DN22008_c0_g1, TRINITY_DN27814_c0_g1, TRINITY_DN22170_c0_g2, TRINITY_DN37163_c0_g1, TRINITY_DN6478_c0_g1, TRINITY_DN4339_c0_g1, TRINITY_DN39736_c0_g1, and TRINITY_DN10302_c0_g1.

In G2, 20 genes and 38 metabolites with significant differences were identified (Figure 5E) including brassinolide, 4-hydroxy-3-methoxycinnamic acid, caffeic acid, chlorogenic acid, D-ribulose, amino-imidazole ribotide, TRINITY_DN31014_c0_g1, TRINITY_DN15303_c0_g1, TRINITY_DN5895_c0_g2, TRINITY_DN2705_c0_g2, TRINITY_DN25686_c0_g2, TRINITY_DN15660_c0_g1, TRINITY_DN6478_c0_g1, and TRINITY_DN3732_c0_g1. In G3, 56 genes and 56 metabolites with significant differences were identified (Figure 5F) including dihydrozeaxin nucleoside, D-glucose, L-cysteine, adenosine-5′-phosphosulfate, TRINITY_DN7358_c0_g1, TRINITY_DN27689_c0_g1, TRINITY_DN7019_c0_g2, TRINITY_DN2374_c0_g1, and TRINITY_DN6652_c0_g1.

### 2.7. Typical Correlation Analysis of the Transcriptome and Metabolome

Correlation analysis in the pentose and glucuronate interconversion pathways revealed that xylitol and ketoglutaric acid were positively correlated with TRINITY_DN9048_c0_g1 and TRINITY_DN18443_c0_g1 while negatively correlated with TRINITY_DN4339_c0_g1, suggesting opposing regulatory roles for these genes in sugar metabolism during early fruit development (Figure S2). In ascorbic acid and alginate metabolism, ketoglutaric acid and 1-phosphorylation-D-galacturonic acid reductase were negatively correlated with TRINITY_DN4339_c0_g1 and TRINITY_DN4091_c0_g1. In glycerol phospholipid metabolism, 3-(O-diphenyl)-SN-glycerol-1-phosphate was positively correlated with TRINITY_DN26505_c0_g1 and negatively correlated with TRINITY_DN10302_c0_g1. In the metabolism of amino sugar and nucleotide sugar during the G2 phase (A vs. B), N-acetamide glycolate and pseudo-amine were significantly positively correlated with TRINITY_DN4587_c0_g1 and TRINITY_DN31014_c0_g1 and negatively correlated with TRINITY_DN5430_c0_g1. In the phenylpropanoid biosynthesis pathway, 4-hydroxy-3-methoxycinnamic acid, caffeic acid, and chlorogenic acid were significantly positively correlated with TRINITY_DN2705_c0_g1 and significantly negatively correlated with TRINITY_DN7957_c0_g1. In the carbon metabolism pathway, ketoglutaric acid, methanamide, gluconic acid, and (3s)-citric acid-CoA were significantly positively correlated with TRINITY_DN25027_c0_g1 and negatively correlated with TRINITY_DN4506_c0_g1.

In G3, within the phenylpropanoid biosynthesis pathway, ferulic acid, 4-hydroxystyrene, and caffeic acid esters showed strong positive correlations with TRINITY_DN2374_c0_g1 while exhibiting negative correlations with TRINITY_DN14357_c0_g1 and TRINITY_DN6652_c0_g1. These opposing correlation patterns highlight candidate genes potentially regulating phenolic compound accumulation in mature LFS fruit. However, the correlation trends of 11 DAMs, including eugenol and coniferyl aldehyde, were the opposite (Figure S2). In glycolysis/gluconeogenesis, D-glucose and oxaloacetate were significantly negatively correlated with eight genes (TRINITY_DN6550_c0_g1, TRINITY_DN24900_c0_g1, etc.). In the carbon metabolism pathway, 5-formyl-5,6,7, 8-tetrahydromethotrexate and succinic acid were significantly positively correlated with the genes TRINITY_DN6550_c0_g1 and TRINITY_DN7543_c0_g1 and significantly negatively correlated with TRINITY_DN5297_c0_g1. The remaining eight DAMs (L-cysteine, citric acid, etc.) all showed significant negative correlations with these three genes. This result lays the foundation for subsequent in-depth studies and the further screening of candidate genes.

Based on correlation patterns, we propose a conceptual model for fruit quality differences between LFS and NT. During G1, differential expression of pentose/glucuronate interconversion genes and metabolites (xylitol, ketoglutaric acid) may modulate cell wall biosynthesis and fruit size. In G2, coordinated changes in phenylpropanoid genes and metabolites (caffeic acid, chlorogenic acid) likely enhance phenolic accumulation and antioxidant capacity in LFS, potentially diverting carbon from sugar accumulation. In G3, starch/sucrose metabolism genes and D-glucose correlations suggest that altered carbon partitioning leads to lower soluble solids in LFS despite larger fruit. This stage-specific network highlights key gene-metabolite modules driving phenotypic divergence.

## 3. Discussion

The ‘Pingguoli’ pear is a common fruit, the growth and development process of which features multiple stages and is accompanied by a series of changes in gene expression and metabolite accumulation. In this experiment, we found that the single-fruit weight of the LFS was significantly greater than that of the NTF, and the mature LFS was 121.25 g heavier than the NTF. The transverse and longitudinal diameters of the NTF and LFS reached 85.8 mm and 67.5 mm and 97 mm and 78 mm, respectively, and the fruits were all flat and round. This finding is consistent with the results of previous studies on the differences in single-fruit weight between the bergamot pear and its various bud mutation varieties [14]. This result may stem from the fundamental differences in the mechanisms that control cell division and cell expansion. During early cell division, the transcriptomic data pertaining to the LFS may involve changes in the expression of core cell cycle regulators, such as cyclins and cyclin-dependent kinases, leading to a higher initial cell number [18]. The results of the analysis of the transcriptome and metabolome data regarding the NTF and LFS indicate that there were 10 important pathways in each developmental period that might be related to the differences in regulating the quality of the NTF and LFS. Among these pathways, the three most important ones are pentose and glucuronide esters; cutin, suberin, and wax biosynthesis; and starch and sucrose metabolism. These pathways have been confirmed to be related to the formation of related traits such as quality and taste during the fruit development process [19,20,21]. In G2, the upregulation of wax biosynthesis genes (e.g., TRINITY_DN5895_c0_g2) likely enhances cuticle deposition, which may improve water retention and maintain turgor pressure during rapid cell expansion, a key driver of fruit enlargement [21]. Enhanced or altered cuticle formation in the LFS could facilitate greater water uptake and retention, thereby promoting more sustained or intense cell expansion, ultimately leading to larger fruit.

Soluble sugar and total soluble solids content are important factors determining the flavor of pears [21]. During fruit ripening, the soluble solids content of the NTF was 15.40%, and that of the NTF was 12.70%. There was also a significant difference in the soluble sugar content between the NTF and LFS. The soluble sugar content of the NTF was significantly higher than that of the LFS, reaching 16.70%. This result is similar to that of previous studies in which it was shown that the soluble sugar content in bergamot pear was significantly higher than that of its bud mutation varieties such as New Pear No. 1, New Pear No. 2, and New Pear No. 6 [22]. The downregulation of sucrose synthase genes (e.g., TRINITY_DN10365_c0_g1) in G3 directly correlates with reduced soluble solids in LFS, suggesting a redirection of carbon from sugar accumulation toward secondary metabolism (e.g., phenolics, flavonoids), which may explain the trade-off between fruit size and sweetness [23]. The significant enrichment of the starch and sucrose metabolism pathway suggests that carbon partitioning is differentially regulated in the LFS. Resources may be preferentially directed towards pathway supporting rapid growth and biomass accumulation (e.g., cell wall synthesis and organic acid metabolism) at the expense of soluble-sugar accumulation in the vacuole [23]. In this experiment, the contents of reducing sugar and soluble protein in the LFS were lower than those in the NTF. This result is extremely similar to prior research results obtained for the bergamot pear and bud mutation varieties [22,24]. The difference between the NTF and LFS may be due to the fact that the large-fruit bud mutation variety is a type of gene mutant, so it may lead to changes in genes related to protein synthesis or stability, thereby affecting the content of soluble protein [25].

In this experiment, the levels of flavonoids and total phenolics in the LFS were higher than those in the NTF, indicating that the NTF and LFS have stronger antioxidant capacity, which is consistent with previous reports [26]. The significant increase in total phenols and flavonoids in the LFS, linked to metabolites like caffeic acid and chlorogenic acid, indicates a shift toward secondary metabolism, which may compete with primary sugar accumulation for carbon skeletons. The altered antioxidant enzyme activities and phenolic profiles in the LFS may also indirectly influence texture by modulating the oxidative cross-linking of cell wall components or the activity of cell-wall-modifying enzymes during maturation [27]. However, the specific mechanism of oxidation still needs to be studied further. After the maturation stage, the ‘Pingguoli’ pear may produce numerous respiratory products and metabolites, including some oxidizing substances, which react with antioxidase, resulting in a decrease in or inactivation of its activity [28]. In this experiment, we found that the activities of superoxide dismutase (SOD) and peroxidase (POD) in the LFS were both higher than those in the NTF. Thus, based on the results above, the overall antioxidant level of the LFS is relatively high, and it has certain antioxidant properties.

The results of the transcriptome sequencing analysis for the LFS and NTF ‘Pingguoli’ pear are consistent with the results of previous studies. The differentially expressed genes mainly correspond to plant hormone signal transduction, fructose and mannose metabolism, the phenylpropane biosynthesis pathway, circadian rhythm in plants, and the glycolysis/gluconeogenesis pathways [29]. These results suggest that the variation observed in the large fruits may generally be due to changes in plant hormones. This finding is similar to the results of this study. In this experiment, through KEGG enrichment analysis, the pathways with the most significant enrichment were determined to be plant hormone signal transduction and the glucose metabolism pathway. The DEGs of the NTF and LFS at each developmental stage were mostly enriched in the secondary metabolite synthesis pathway, and the number of enriched DEGs showed a trend of upregulation at the developmental stage.

A total of 4910 metabolic compounds were identified, including caffeine, gynostemma pentaphyllum saponin A, glucolactone, gluconic acid, neopentolactone D, and narbomycin. Through KEGG enrichment analysis, we found that in the three stages of fruit development, the DAMs of the NTF and LFS were enriched in the photosynthesis, diterpene biosynthesis, phosphate inositol metabolism, pentose/glucuronate interconversion, and cutin/wax biosynthesis pathways. These critical pathways affect the differences in quality traits between the NTF and LFS. The enrichment of pathways related to pentose/glucuronate interconversion and cutin/wax biosynthesis points to modifications in hemicellulose, pectin, and cuticular layers [21,30]. Changes in pectin metabolism can directly affect middle lamella cohesion and cell–cell adhesion, impacting fruit firmness and mealiness [30]. Therefore, the bud mutation appears to have triggered a coordinated yet divergent reprogramming of gene networks governing cell growth, primary carbon metabolism, and secondary phenolic biosynthesis, collectively shaping the distinct size–quality–texture phenotype of the LFS relative to the NTF.

In this experiment, further analysis was conducted in combination with an analysis of DEGs and DAMs in the relevant pathways, and a total of 98 DEGs and 125 DAMs were screened out. Among them, the expression of 42 DEGs was upregulated, and that of 56 DEGs was downregulated. Regarding content accumulation, 57 of the DAMs were upregulated and 68 were downregulated. Correlation network and typical correlation analyses were conducted on the DEGs and DAMs to further explore the key genes most related to the metabolites. The results indicate that during cell division (G1), the NTF and LFS screened out a total of seven key genes. Seven key genes were also screened out during the rapid growth phase (G2). A total of 79 key genes were screened out during the mature stage (G3). Among these key genes, there may be some that regulate the large-fruit bud transformation of the ‘Pingguoli’ pear and affect quality. These results provide a theoretical basis for subsequent in-depth research on the functions of these genes.

## 4. Materials and Methods

### 4.1. Plant Materials

In this experiment, samples of normal-type fruit (NTF) of the ‘Pingguoli’ pear and large-fruit bud sport lines of *Pyrus pyrifolia* cv. Pingguoli (LFS) were taken from the Fruit Research Institute of the Yanbian Korean Autonomous Prefecture Academy of Agricultural Sciences (5 × 6 m 11-year-old trees, with an area of 2 hm^2^). After many years of continuous observation in this experiment, we found that the average fruit of the LFS ‘Pingguoli’ pear reached more than 250 g, reflecting a large fruit, and had stable traits. In this experiment, based on the local climatic conditions and different developmental stages of the fruits, two types of ‘Pingguoli’ pears from the NTF and LFS varieties were collected at 30 days (cell division stage, G1), 70 days (rapid growth stage, G2), and 120 days (maturity stage, G3) after the full-bloom stage (Figure 1). For each group, fruits of the same size and free from diseases and pests were picked at different stages (>10). Three biological replicates were set for each variety. The parts of the ‘Pingguoli’ pear samples were immediately rapidly frozen in liquid nitrogen and stored at −80 °C for transcriptome and metabolome detection. Another part of each ‘Pingguoli’ pear sample was put into an icebox and taken back to the laboratory for the determination of physiological indicators.

### 4.2. Determination of Appearance Quality and Soluble Solids Content

The weight of a single fruit of the ‘Pingguoli’ pear was measured using an electronic balance (China Shunyu Hengping Instrument FA2004, Shanghai, China) (accurate to 0.01 g). The transverse and longitudinal diameters of the ‘Pingguoli’ pear fruits were measured with a Vernier caliper (M33×2-6H) (China DeLi DL92300B, Ningbo, China) (accurate to 0.01 mm). The soluble solids content of the ‘Pingguoli’ pear fruits was measured using a handheld refractometer (Japan ATAGO MASTER-20PT, Tokyo, Japan). Three biological replicates were set for each group.

### 4.3. Determination of Reducing Sugar and Soluble Sugar Content

The content of reducing sugar was determined using the 3, 5-dinitrosalicylic acid method. A total of 1.0 g of ‘Pingguoli’ pear pulp was weighed and ground in a mortar and then transferred into a 25 mL tube. Distilled water was added to the tube up to the mark. The tube was kept in a constant-temperature water bath at 80 °C for 30 min to allow extraction of the reducing sugar. After cooling, the remaining substances were rinsed with 20 mL of distilled water, and the two filtrates were collected in 100 mL volumetric flasks until the volume reached the calibration line to obtain the reducing sugar extract. We then took 25 mL graduated test tubes and added 2.0 mL of reducing sugar extract and 1.5 mL of 3, 5-dinitrosalicylic acid reagent, respectively. We measured the absorbance (OD) at a wavelength of 540 nm and calculated the content of reducing sugar.

We weighed 1.0 g of ‘Pingguoli’ pear flesh, ground it, and poured it into a marked test tube. We added 6 mL of distilled water, covered the opening of the tube with plastic film, and boiled it for 30 min. After cooling, filtering was performed, and the volume was made up to 100 mL. We collected the filtered liquid in a test tube and then added 6 mL of distilled water and boiled it for 10 min. After re-filtering, we made up the volume to 100 mL. We then took 0.5 mL of the sample extract and 1.5 mL of distilled water, added 0.5 mL of anthranone-ethyl acetate and 5.0 mL of concentrated sulfuric acid, shook the mixture thoroughly, and kept it warm in a boiling water bath for 1 min. Finally, the absorbance (OD) was measured at a wavelength of 630 nm, and the content of soluble sugar was calculated.

### 4.4. Determination of Soluble Protein, Total Flavonoids, and Total Phenol Contents

A total of 2.0 g of ‘Pingguoli’ pear flesh was weighed and ground with 5 mL of distilled water. This was then centrifuged at 12,000 *g* rpm for 20 min to remove impurities. The supernatant was collected and stored at low temperature. We took 1.0 mL of the supernatant and 5.0 mL of Coomassie Brilliant Blue G-250 solution, mixed them well, and let the mixture stand for 2 min. The absorbance (OD) was measured at a wavelength of 595 nm, and the content of soluble protein was calculated.

The ‘Pingguoli’ pear flesh was dried at 50 °C until it reached a constant temperature and then ground into powder and passed through an 80-mesh sieve. We weighed 0.1 g of the dry powder sample, put it into a 2 mL centrifuge tube, and then added 2 mL of 70% ethanol solution. We performed ultrasonic extraction on the configured centrifuge tubes (60 °C, 30 min). We also centrifuged after removal (3000 rpm, 30 min). We then obtained the supernatant and diluted it twice. Then, we took 0.5 mL and added 2 mL of ethanol. We then added NaNO_2_ and let the mixture stand for 6 min. Then, we added Al(NO_3_)_3_ and let the mixture stand for 6 min. Finally, we added NaOH and let the mixture stand for 10 min. Absorbance (OD) was measured at a wavelength of 510 nm, and the content of total flavonoids was calculated.

A 0.1 g dry powder sample of the above-mentioned ‘Pingguoli’ pear was weighed and added to 2 mL of distilled water in a tube; this was followed by ultrasonic extraction (60 °C, 30 min). Centrifugation was applied after removal (3000 rpm, 30 min). We then obtained the supernatant and diluted it 5 times. Then, we took 0.2 mL of the extract; added 5 mL of distilled water, 1 mL of foline-phenol, and 3 mL of Na_2_CO_3_, respectively; and shook the mixture for 1 h. Absorbance (OD) was measured at a wavelength of 760 nm, and the content of total phenols was calculated.

### 4.5. Determination of Antioxidant Enzyme Activity

The activity of SOD was determined using the nitrogen tetrazolium staining method [31]. The activity of POD was determined using the guaiacol method [32]. We took 50 mL of phosphate-buffered solution (PH = 6.0, 100 mmol/L), added 28 μL of methyl catechol, and heated and stirred the mixture. After it cooled, we added 19 μL of 30% H_2_O_2_, mixed it well, and stored it in the refrigerator for refrigeration and later use. We then took 0.5 g of the pulp and placed it in a pre-cooled mortar. We added 1 mL of phosphate-buffered solution with a pH 6.0, ground it into a pulp, and centrifuged it at 4000 *g* rpm for 15 min, retaining the supernatant. At 470 nm, we took a cuvette, added 3 mL of methyl catechol mixture and 1 mL of enzyme solution, immediately started timing with a stopwatch, took a reading once every 1 min for a total of 4 times, and calculated the POD activity by increasing the amount of CD470 by 1 enzyme activity unit.

### 4.6. Transcriptome Sequencing and Analysis

In this experiment, the extraction of total RNA from the Pingguoli samples used for sequencing and the construction of libraries were both accomplished by Beijing Biomarker Technologies. Eighteen libraries were constructed during three different periods of ‘Pingguoli’ pear development, namely, the NTF fruit’s cell division phase (G1 phase)—G1Z1, G1Z2, and G1Z3; the rapid growth phase (G2 phase)—G2Z1, G2Z2, and G2Z3; and the cell maturity phase (G3 phase)—G3Z1, G3Z2, and G3Z3. For the LFS fruit, the cell division stage (G1 phase) corresponded to G1Y1, G1Y2, and G1Y3; the rapid growth phase (G2 phase) corresponded to G2Y1, G2Y2, and G2Y3; and the maturity stage (G3 phase) corresponded to G3Y1, G3Y2, and G3Y3. Three biological repetitions were employed in each stage.

The Trinity (R14.1.3) software extends the small, shorter fragments (K-mer) into longer fragments (Contig) and utilizes the overlap between these fragments to obtain the fragment set (Component) [33]. Using the De Bruijn plot method and sequencing Read information, we identified transcript sequences in each fragment set. The unigene sequence was compared with the NR, Swiss-Prot, COG, KOG, eggNOG4.5, and KEGG databases using the DIAMOND (Version 5.1) software [31,32]. The KEGG Orthology results of Unigene in KEGG were obtained using KOBAS (Version 2.0), and the GO Orthology results of new genes were analyzed using the InterPro integrated database via InterProScan (Version 5.70-102.0) [32,33,34,35]. After predicting the amino acid sequence of Unigene, the HMMER (Version 3.1) software was used for comparison with the Pfam database to obtain the annotation information for Unigene [35,36,37]. The analysis of differentially expressed genes (DEGs) was performed using the DESeq2 (Version 1.48.1) software [37,38]. Fold change ≥ 1.5 and FDR < 0.05 were used as screening indicators for verification. The fold change represents the expression level between two pairs of samples (groups). For the calculation of the difference multiple, the logarithmic value is used, which is represented as log2 FC. The larger the absolute value of log2 FC and the smaller the FDR value of the gene, the greater the degree of difference in the change in the two groups of samples. Three biological repetitions were used in each stage.

### 4.7. Metabolome Detection

We weighed 50 mg of the Pingguoli sample, added 1000 μL of the extract containing the internal standard (1000:2) (the volume ratio of methanol, acetonitrile, and water was 2:2:1, and the internal standard concentration was 2 mg/L), and stirred the product with rotation for 30 s. We then added porcelain beads, ground them with a 45 Hz (10 min) grinder, and performed ultrasonic treatment (ice water bath) for the same amount of time. We then kept the sample at −20 °C for 1 h and centrifuged it at 4 °C and 12,000 *g* rpm for 15 min. We then extracted 500 μL of the supernatant and placed it in an EP tube. After the extract was dried in a vacuum concentrator, we added 160 μL of the extract (acetonitrile/water ratio = 1:1) to the metabolites and redissolved them until dried metabolites were obtained. We continued rotation and stirring for 30 s and then performed ultrasonic treatment for 10 min (in an ice-water bath). The sample was centrifuged at 12,000 *g* rpm at 4 °C for 15 min. We then took 120 μL of the supernatant and placed it in a 2 mL syringe. We withdrew 10 μL samples to prepare quality control samples for testing [39]. Chromatographic conditions: Metabolomic analysis was conducted using a combination of ultra-high-performance liquid chromatography (Acquity I-Class PLUS) and high-resolution mass spectrometry (Xevo G2-XS QTof) (all from Waters, Milford, MA, USA). Mass-spectrometry conditions: High-resolution mass spectrometers (Waters, Xevo G2-XS QTof) were used to collect primary and secondary mass-spectrometry data in MSe mode. In each data acquisition cycle, both low-collision and high-collision energies of the two channels could be achieved simultaneously (low-collision energy, 2 V; high-collision energy range, 10–40 V; scanning frequency, 0.2 s). Three biological replicates were set for each group.

### 4.8. Data Analysis

All of the test data in this study were analyzed using Excel 2021. One-way ANOVA and regression analysis were performed using SPSS v27.0. The original data collected via MassLynx v4.2 were processed using the Progenesis QI software (Version 2.4) for peak extraction, peak alignment, and other data-processing operations. The online METLIN database and the self-built database of Beijing Biomarker Technologies were scanned using the Progenesis QI software. On this basis, the identification results of theoretical fragments were verified. The error was less than 100 ppm. All data are presented as the mean ± standard deviation (SD) from three biological replicates. Statistical significance was determined by the Student’s *t*-test, with *p* < 0.05 considered significant.

## 5. Conclusions

In this experiment, by analyzing the dynamic characteristics of the fruit development of the large-fruit-type bud mutation ‘Pingguoli’ pear, we found that its single-fruit weight was significantly greater than that for the NTF. The differentially expressed genes and differentially accumulated metabolites in the NTF and LFS were mostly enriched in the secondary metabolite synthesis pathways. The findings indicate that the significant increase in fruit size in the LFS can not only be attributed to the coordinated regulation of cell division and expansion during critical phases (G1 and G2), but is also closely linked to a comprehensive reprogramming of specific metabolic pathways. During the early fruit development stage (G1), the bud mutant modulates the pentose and glucuronate interconversion pathway, influencing cell wall composition and structure, thereby laying the foundation for cell expansion. In the rapid fruit expansion phase (G2), the significant enrichment of the plant hormone signal transduction pathway suggests that altered dynamic balance of hormones such as auxin and gibberellin plays a central role in driving sustained cell enlargement. By the fruit maturation stage (G3), differences in the starch and sucrose metabolism pathway are directly associated with the reduction in soluble sugar content and the marked increase in secondary metabolites such as phenols and flavonoids, explaining the observed trade-off between “fruit enlargement” and “reduced sugar but enhanced phenol content” in the large-fruit mutant. Additionally, the key genes screened, such as TRINITY_DN6550_c0_g1, provide a basis for the subsequent in-depth study of key genes. In the future, researchers could employ gene editing or transgenic technologies to functionally validate the screened key candidate genes, thereby clarifying their causal roles in fruit size and quality formation.

## Data Availability

The raw data supporting the conclusions of this article will be made available by the authors on request.

## Supplementary Materials

The following supporting information can be downloaded at: https://www.mdpi.com/article/10.3390/plants15081225/s1.
