# Supplementary material for: Combined Transcriptome and Metabolome Analysis of the Quality Change Mechanism of the ‘Pingguoli’ Pear with a Large-Fruited Bud Mutation
Source: Plants (Basel). 2026 Apr 16;15(8):1225. doi: 10.3390/plants15081225 (PMC13119805; doi:10.3390/plants15081225)
Supplement: Supplementary file 1 [file plants-15-01225-s001.zip › plants-4122644-supplementary.pdf]

Additional Figures

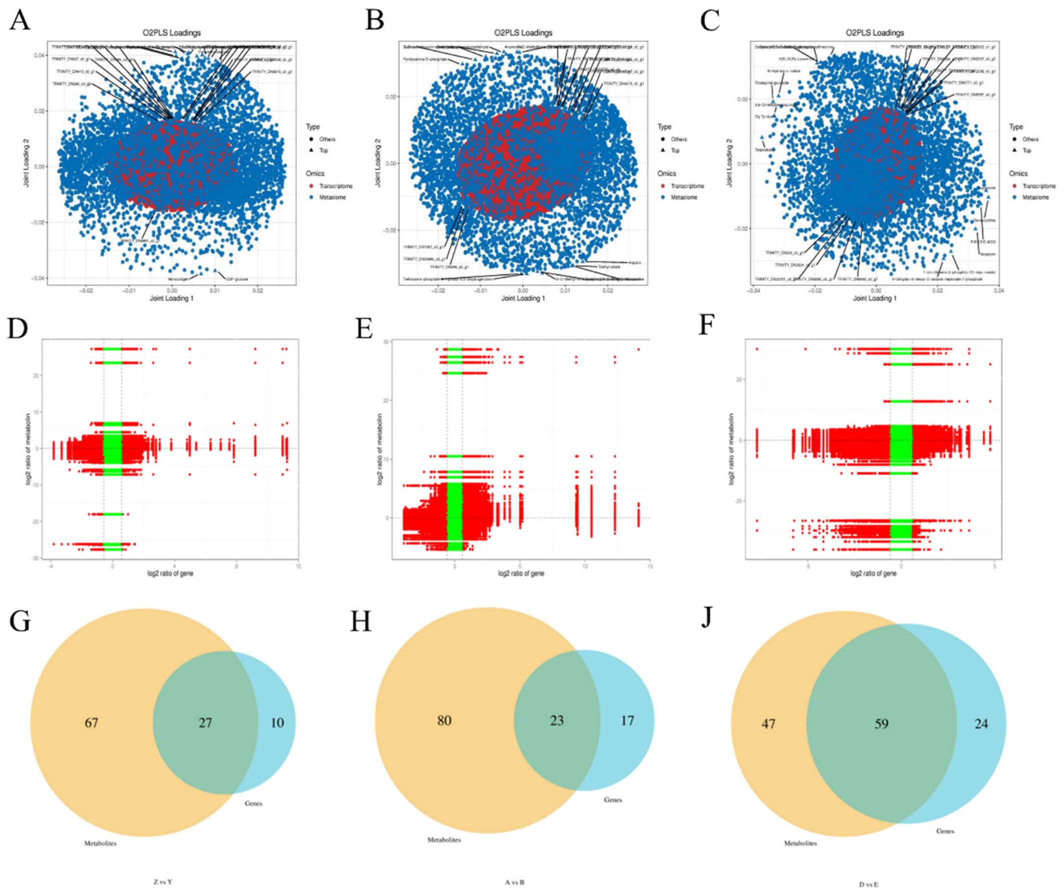

Figure S1. (A) Loading plots of transcriptome and metabolites of the O2PLS model of G1Z vs G1Y. (B) Loading plots of transcriptome and metabolites of the O2PLS model of G2A vs G2B. (C) Loading plots of transcriptome and metabolites of the O2PLS model of G3D vs G3E. (D) Nine quadrant diagram for correlation analysis of G1Z vs G1Y. (E) Nine quadrant diagram for correlation analysis of G2A vs G2B. (F) Nine quadrant diagram for correlation analysis of G3D vs G3E. (G) Venn analysis of G1Z vs G1Y. (H) Venn analysis of G2A vs G2B. (J) Venn analysis of G3D vs G3E.

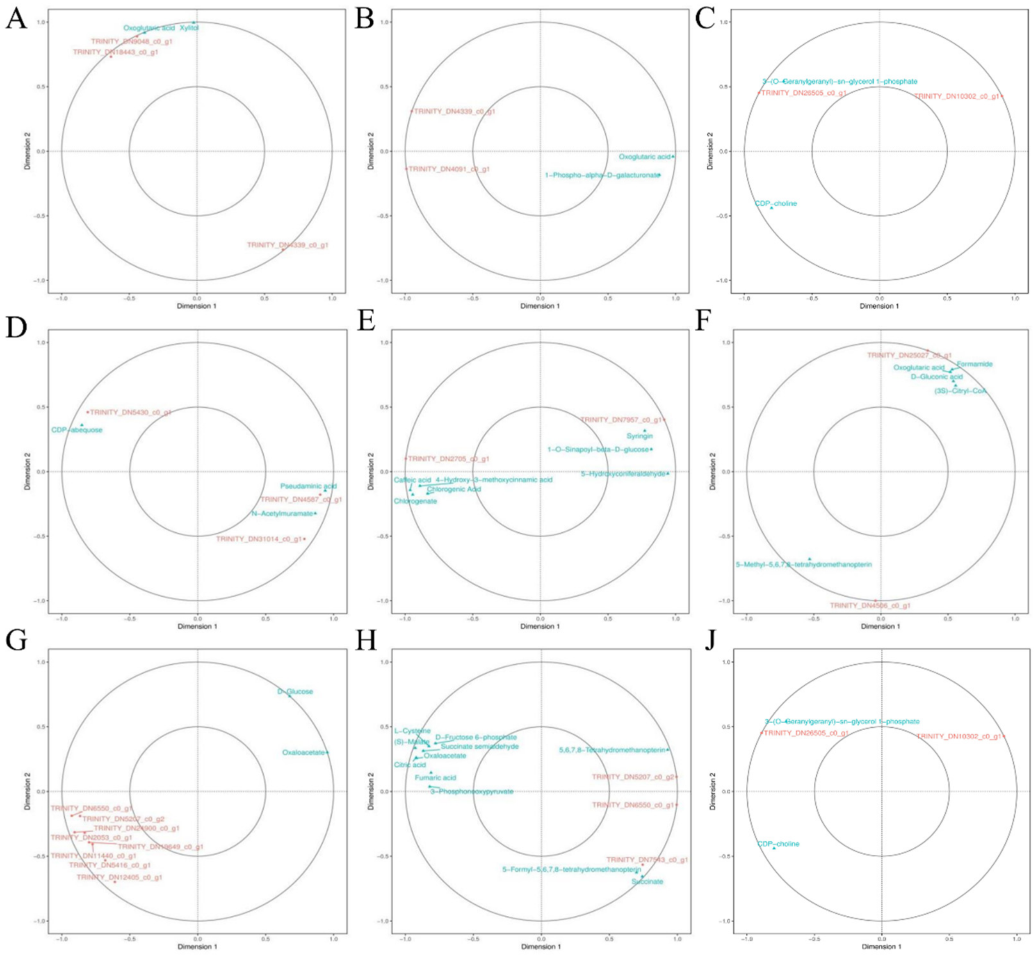

Figure S2. Typical correlation analysis plots. Image A1-A3 is G1Z vs G1Y、B1-B3 is G2A vs G2B、C1-C3 is G3D vs G3E.

Table S1:

Table S1. Statistical table of sample sequencing data evaluation.

Sample Number	Read Number	Base Number	GC Content/%	%≥Q30
G1Y1	20,545,046	6,154,679,399	46.82%	93.59%
G1Y2	20,340,752	6,091,875,417	46.92%	95.69%
G1Z1	20,028,316	5,998,951,328	46.75%	92.50%
G1Z2	21,532,165	6,445,455,514	46.80%	93.34%
G1Z3	19,995,471	5,990,872,447	46.79%	93.65%
G2Y1	20,366,468	6,096,164,994	46.63%	93.45%
G2Y2	19,520,108	5,847,729,149	46.66%	93.40%
G2Y3	20,392,269	6,109,247,119	46.99%	93.57%
G2Z2	21,472,088	6,432,356,906	46.80%	93.78%
G2Z3	20,362,139	6,099,688,604	46.62%	93.37%
G2Z1	19,495,390	5,840,725,735	46.70%	93.33%
G3Y1	20,783,159	6,226,673,377	46.89%	93.24%
G3Y2	19,770,057	5,992,257,725	46.96%	92.96%
G3Y3	22,563,484	6,749,886,412	46.80%	93.78%
G3Z1	20,247,891	6,066,124,693	46.94%	93.19%
G3Z2	20,538,930	6,149,691,713	46.97%	96.04%
G3Z3	21,413,285	6,413,310,188	46.87%	93.72%
G1Y3	22,315,659	6,686,191,465	46.81%	93.32%
